# The effect of three types of water-based training protocols on thymus atrophy and specific indicators of cellular immune senescence in aged male rats

**DOI:** 10.1007/s10522-025-10183-5

**Published:** 2025-01-20

**Authors:** Mohammad Jahan-Mahin, Roya Askari, Amir Hossein Haghighi, Omid Khaiyat

**Affiliations:** 1 Department of Exercise Physiology, Faculty of Sport Science, Hakim Sabzevari University, Sabzevar, Iran; 2 School of Health and Sport Sciences, Liverpool Hope University, Liverpool, UK

**Keywords:** Exercise, Aging, Aquatic, Resistance, Endurance, Immune senescence, Thymus

## Abstract

The collective detrimental impact of aged naive lymphocytes and thymus atrophy on the aging of the immune system can be mitigated by exercise. Hence, this research aims to explore the effects of three methods of water-based exercises on immune system aging and thymus atrophy in elderly rats. Thirty-two 24-month-old rats, with an average weight of 320 ± 5 g, were randomly allocated into four groups of endurance training (n = 8), resistance training (n = 8), combined training (n = 8), and control (n = 8).The training protocols (10 weeks) were conducted four times a week in a container measuring 50 × 50x100 cm filled with water at 30 ± 1 °C. The evaluation of naïve and memory T lymphocytes was conducted for the intervention groups based on the expression or lack of expression of the CD28 and CD57 markers in the subsets of CD4 + and CD8 + T cells. Naïve T cells were represented by CD28 + CD57- T lymphocytes, memory T cells were represented by CD28- CD57- T lymphocytes, aged naïve T cells were indicated by CD28 + CD57 + lymphocytes, and aged memory T cells were represented by CD28- CD57 + lymphocytes. The findings of the study showed that all three exercise protocols resulted in a significant decrease in levels of memory CD8, aged CD8, naive and naive CD4 and CD8, and aged memory, as well as an increase in levels of CD4, CD8, CD4 + , and naive CD8 when compared to the control group. It was observed that thymus atrophy, memory CD4, and aged CD4 had a significant decrease only in the combined exercise group compared to the control group, with no significant differences observed in these indicators for the resistance and endurance groups. Furthermore, the ratio of CD4 to CD8 remained unchanged across all groups. The findings of this study suggest greater efficacy of combined training in enhancing specific health indicators of cell immunity among elderly populations. Moreover, engaging in water exercises of all three types of combined, resistance, and endurance training are deemed safe activities for older individuals to bolster their immune system and mitigate the aging process of T cells.

## Introduction

Aging is associated with the modulation of both components of the immune system arm (Witkowski et al. 2022). As individuals age, naive T cells gradually transition to distinct phenotypes of memory and senescence. On the other hand, the process of atrophy of the thymus gland progresses rapidly until middle age, with only about 15% of the thymus tissue function remaining by age 50. This reduction leads to a further decrease in naïve T lymphocytes (Palmer 2013). Although there are several possible reasons for the aging of the immune system, the main factors contributing to this phenomenon include a diminished population of naïve lymphocytes, degeneration of the thymus, and accumulation of aged T cells in particular. This condition is primarily caused by the accumulation of aged T cells associated with aging. Aged T cells are dysfunctional immune cells that are alive but cannot divide, resist apoptosis, and tend to produce increased levels of pro-inflammatory substances and matrix-degrading enzymes (Deursen 2014; Yuan et al. 2018). These factors increase older people's mortality vulnerability (Cao Dinh et al. 2019).

Lifestyle interventions can help maintain the natural aging process and prevent premature aging of the immune system. Among these interventions, diets and exercise are the primary non-pharmacological strategies to combat age-related immune decline (Akunuru and Geiger 2016). It has been shown that exercise training causes transient changes in immune responses at rest and in response to physical activities (during the recovery period). Based on previous studies, long-term exercise can be a safe intervention to prevent low-grade chronic inflammation and aging of the immune system (Deursen 2014; Valdiglesias et al. 2017).

Exercise can be described as a form of "immunotherapy" which is highly cost-effective and can significantly enhance the quality of human life (Araújo et al. 2013). In this context, Merellano-Navarro et al. (2021) noted that active elderly individuals possess more naive T lymphocytes than their inactive counterparts. While mechanism by which exercise enhances the immune system is not fully understood, increase in the production of free radicals has been suggested as potential factor (Merellano-Navarro, et al. 2021). During exercise, oxygen consumption increases up to 10 times, significantly increasing free radicals leading to the immune system to become more effective at combating harmful free radicals in the blood. This increased production of antioxidant enzymes, such as superoxide dismutase, catalase, and glutathione peroxidase, helps regulate antioxidant enzyme activity, enhances cellular immune response, and boosts the numbers of CD4 + and CD8 + cells (Finaud et al. 2006).

Moderate-intensity exercise can offset the age-related decrease in sympathetic system function and beta-adrenergic receptor sensitivity and lead to increased catecholamine secretion, stimulation of the spleen, lymph nodes, thymus, and lymph cells, and proliferation of T and CD4 + cells to compensate for the decline in CD8 + cells. Stimulation of beta-adrenergic receptors may also activate cAMP production in lymphocytes (Abd El-Kader and Al-Shreef 2018). Both endurance and resistance exercises are advocated as anti-inflammatory treatments with the potential to enhance immune function. (Ploeger et al. 2009) The role of endurance training in enhancing quality of life with aging has received a high attention over the past decade. Endurance training is being increasingly utilized to improve functional abilities and perfromance of older adults during daily activities. Particular studies on endurance training have demonstrated enhancements in the immune system of elderly individuals. Woods et al. (2003) found that enduring moderate-intensity training (speed 13–22 m/min) for four months led to changes in naive and memory cells in TCD4 + and TCD8 + lymphocytes in the spleen tissue of mice. The study conducted by Brito-Neto et al. (2019) revealed that 12 weeks of resistance training, with an intensity rate of 6–7 on the Borg index, resulted in a significant rise in the count of TCD4 + lymphocytes in AIDS patients. (Ploeger et al. 2009).

Compared to research on immune responses to either endurance or resistance exercises, limited studies have investigated the impact of combined (concurrent) training protocols. In this context, Despeghel et al. (2021) reported a promising improvement in signs of immune system aging following six weeks of combined training (comprising six stations at 60% of 1RM for resistance and 20 min of cycling for endurance training) in a sample of 70-year-old individuals even within a short period and at a low threshold. Building on the initial findings, it is crucial to further explore which training types influence the aging process of the body’s immune system in a more effective way (Despeghel et al. 2021). Water-based exercise is considered as a rigorously controlled training protocol. Studies have shown that water exercise can delay tumor growth (Almeida et al. 2009) and enhance mice's anti-inflammatory and neuroprotective effects (Bernardes et al. 2013). Additionally, when compared to dry land exercise, this type of exercise can enhance support for the immune and nervous systems (Deforges et al. 2009; Goes et al. 2014). On the other hand, it has been observed that the physiological responses to long-term immune adaptations to exercise depend on the type and dosage of exercise (Campbell and Turner 2018; Kakanis et al. 2010).

Although water-based exercise is recommended for older people, few studies have been conducted to evaluate its effects on the immune system. Xie et al. (2019) found that high-intensity swimming training, with a weight equal to 4% of the rat's body weight, compared to moderate-intensity training without weight, performed for 50 min, five days a week for six weeks, resulted in a decrease in inflammatory markers and an increase in anti-inflammatory markers in mice with multiple sclerosis (Xie et al. 2019). However, both intensities showed no significant difference in CD4 + and CD8 + T lymphocyte levels. This study suggested that by changing the intensity and duration of exercise training along with the increasing load during the training period, the frequency of TCD4 + and TCD8 + lymphocyte subsets in the lymphatic organs can be better adjusted. According to Meneguello-Coutinho et al. (2014) a swimming training protocol with loads equal to 2% of body weight, performed five days a week for six weeks (1-h/session), reduced the amount of lymphocyte proliferation in the training group compared to the controls (Meneguello-Coutinho et al. 2014). Furthermore, Morgado et al. (2018) observed an increase in the levels of leukocytes, total lymphocytes, CD4 + , CD8 + , and CD3 + following an incremental swimming session (7 repetitions of 200 m) in young men, (Morgado et al. 2018). The study also reported a decrease in the ratio of CD4 + /CD8 + due to elevated CD8 + expression, which was linked to the rise in natural killer (NK) cells post-exercise.

Research on the effects of swimming training on the immune system has primarily focused on multiple sclerosis patients (Xie et al. 2019) and athletes (Kwon et al. 2005; Morgado et al. 2020) assessing immunoglobulins and interleukins (Kapilevich et al. 2017). Considering the safety of water exercises for older adults and the lack of studies investigating combined endurance and resistance water exercises on naive and aged T-lymphocyte indices, this study aims to examine the effects of three types of water exercises on relevant indices in healthy old rats.

## Methods

The present study is an experimental-developmental research with a post-test design involving elderly male Wistar field rats. Thirty-two rats with an average age of 12 ± 1 month were procured through appropriate processes and underwent the aging process (reaching the age of 24 months) prior to partaking in 10-week exercise training regimens. The study received required ethical approval from the institutional (Hakim Sabzevari University) board (IR.HSU.AEC.1401.003). The samples were randomly divided into four groups (8/group) including three exercise groups of endurance, resistance, and combined, and one control group. Throughout the study, all rats were housed in the same environment with an average temperature of 23 ± 3 °C, humidity level of 50 ± 10%, and a light–dark cycle of 12–12. They were provided with ad libitum access to food and water. After a week of adapting to the environment, a training familiarization period was completed in line with the training protocols. After the familiarization training period, two rats from the control and endurance training groups and one rat from the combined and resistance groups passed away. Ultimately, 6 rats from the control and endurance groups and 7 rats from the combined and resistance groups were included in the evaluation.

### Resistance training protocol

This training was performed four times a week for 10 weeks in an aquarium measuring 50 × 50x100 cm, with water temperature maintained at 30 ± 1 °C. The animals were introduced to resistance exercises over two weeks, with five sessions per week. In the familiarization session, rats were placed in a container filled with water where they carried loads equivalent to 10% of their body weight in the first session, 15% in the second and third sessions, and 20% in the fourth and fifth sessions. A mesh metal net, similar to the rat resistance training ladder with 2 cm spacing between each mesh (Dai et al. 2020), was attached to the container wall as a climbing apparatus. Submerged in water, the rats were required to climb up a ladder for the exercise. During the familiarization week, exercises consisted of three sets of 8 repetitions, with a 1 min rest between the sets.

In the first session of the familiarization week, the rats were released close to the ladder to allow minimal immersion before climbing. In the following sessions, the release distance was gradually increased determined by an intermediary. During the main training period the water level was set at approximately 200% of the animal's body length. The rats were then released into the water from a distance of 35 cm to reach and climb up the ladder. The selection of this distance was determined based on the time the animal could spend underwater without hypoxia, ensuring it did not exceed 10 s (Lima et al. 2013). These procedures were conducted in 4 sets with ten repetitions each, with a 1 min rest interval between sets (refer to Table 1). Using a band, the load weights were attached to the beginning of the rats' tails.

**Table 1:** Resistance training protocol

Adaptation weeks (2 week–5 sessions/week)	
Day 1 Session	Weight: 10% of the body weight, 3 sets of 8 repetitions, 1 min rest interval between sets, water height equal to 100% of the rat's length
Day 2&3 Sessions	Weight: 15% of the body weight, 3 sets of 8 repetitions, 1 min rest interval between sets, water height equal to 120% of the rat's length
Day 4&5 Sessions	Weight: 20% of the body weight, 3 sets of 8 repetitions, 1 min rest interval between sets, water height equal to 140% of the rat's length

Resistance training weeks (10 weeks)	Amount of weight based on body weight	Rest between each set (min)	Repetition	Set
1&2	30%	1	10	4
3&4	35%	1	10	4
5&6	40%	1	10	4
7&8	45%	1	10	4
9&10	50%	1	10	4

### Endurance training protocol

Similar to the resistance group, familiarization sessions in the endurance group were also conducted over five sessions per week. On the first day, the animals swam for 5 min in water at a height equal to 100% of their body length, without weights. On the second and third days, the animals swam for 10 min in water at a height equal to 120% of their body length. On the fourth and fifth days, they swam for 15 min with the water set at a height equal to 140% of their body length. This height was maintained throughout the rest of familiarization period. Then, in the subsequent 10 weeks the rats carried out the main endurance training program (Table 2) (Xie et al. 2019). The container was divided into two sections (measuring 25 × 100 cm) for endurance training, allowing each rat to swim separately in its lane (Fig. 2).

**Table 2:** Endurance training protocol

Adaptation week (one week)	
Day 1 Session	Swimming for 10 min with a water height of 100% of the rat's body length
Day 2&3 Sessions	Swimming for 15 min with a water height of 120% of the rat's body length
Day 4&5 Sessions	Swimming for 20 min with a water height of 140% of the rat's body length
**Endurance training weeks (10 weeks)**	**The duration of the exercise**
1&2	Continuous swimming for 30 min
3&4	Continuous swimming for 35 min
5&6	Continuous swimming for 40 min
7&8	Continuous swimming for 45 min
9&10	Continuous swimming for 50 min

### Combined training protocol

This training was conducted four times a week for 10 weeks alternately, with one day dedicated to endurance training and the next to resistance protocols. In relation to the control group, each rat was placed freely in a container with a water depth of 5 cm four sessions per week (30 min/session) to ensure similar physiological stress conditions with the exercise training groups (Altarifi et al. 2019).

### Immunohistochemistry

The animals were anesthetized with chloroform and dissected 48 h after the last training session and the spleen tissue was stored in 10% formalin for 72 h. The next steps involved removing water from the tissue using 70% alcohol, application of xylene to eliminate the alcohol, and introducing paraffin to the tissue to solidify and prepare it for shaping and sectioning.

The samples were sliced to a thickness of 5 µm using a microtome, positioned on a salinized slide, and slides were immersed in TBS 1X solution (T5912-Sigma) inside a microwave. The microwave was switched off once the solution reached its boiling point with the samples left in the solution for 20 min. Subsequently, the samples underwent a thorough washing process involving PBS (P4417—Sigma) in three sequential steps, each with a 5-min interval. Next, 0.3% Triton X-100 was introduced to the samples for 30 min to facilitate cell membrane permeation. The samples were subsequently rinsed with PBS, and 10% goat serum was added to block the secondary antibody reaction for 45 min. The primary antibodies CD4 (orb4830), CD8 (orb323288), CD28 (orb378206), and CD57 (orb385450) (Biorbyt, Cambridge, UK) were diluted 1 to 100 with PBS and applied to the samples.

Samples were then placed in a humid environment in a refrigerator at a temperature of 2 to 8 °C for 24 h to prevent tissue desiccation. After 24 h, the container with the tissue was removed from the refrigerator and washed four times with PBS for 5 min each. Subsequently, the secondary antibody was added at a 1:150 dilution and incubated in darkness for 1 h and 30 min in a 37 °C incubator (AriaTeb, Iran). The sample was moved from the incubator to a dark room, followed by three washes. DAPI (D9542-Sigma) was then added to the samples. After 20 min, the samples were rewashed with PBS. Finally, a mixture of glycerol and PBS solution was applied to the sample, and a coverslip was used for fluorescent photography with an Olympus microscope. The presence or absence of CD28 and CD57 markers in CD4 + and CD8 + cells was utilized to differentiate between naïve T lymphocytes (- CD57 + CD28) and senescent naïve T lymphocytes (CD28 + CD57 +).

### Statistics

The normality of data distribution was assessed using the Shapiro–Wilk test, while the homogeneity of variances was evaluated with Levine's test. To compare the research indicators in between training groups, the data were analysed using one-way ANOVA and Kruskal–Wallis statistical methods at the significance level of P ≤ 0.05 using SPSS version 22 software.

## Results

**Fig. 1:**
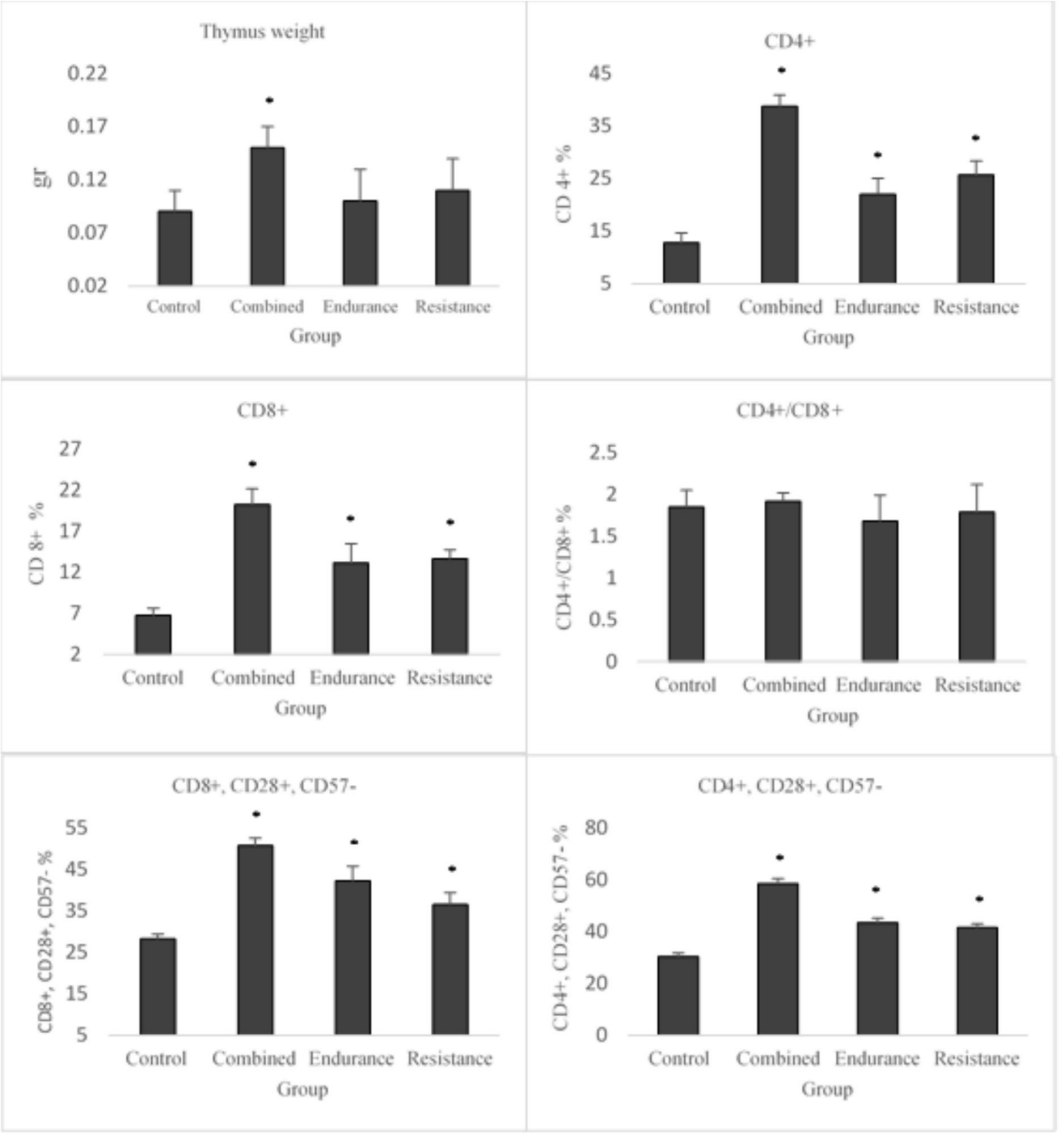
Training-induced changes in the percentage counts of Thymus weight, naïve and memory phenotype between group. (*) Difference with the control group at the P ≤ 0.05 level

**Fig. 2:**
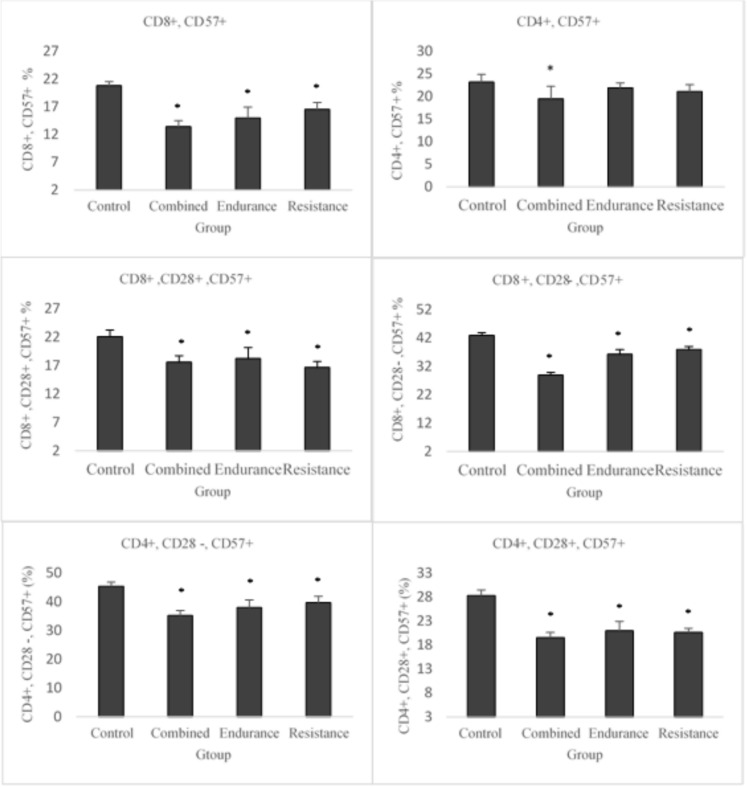
Training-induced changes in the percentage counts of senescent phenotype between group. (*) Difference with the control group at the P ≤ 0.05 level

Main results are presented in Table 3, Fig. 1 and Fig. 2. Results indicate that all interventions including resistance, endurance, and combined training led to a significant decrease in indicators associated with aged TCD8 + (CD8 + , CD57 +), naive TCD8 + (CD8 + , CD28 + , and CD57 +), and memory-aged (CD8 + , CD28-, and CD57 +), as well as pristine TCD4 + (CD4 + , CD28 + , and CD57 +) and memory-aged (CD4 + , CD28-, and CD57 +).

**Table 3:** Comparisons of the study variables across the exercise training groups

Variables	Control	Resistance training	Endurance training	Combined exercise
	mean ± SD	mean ± SD	mean ± SD	mean ± SD
Thymus weight	0.09 ± 0.02	0.11 ± 0.02	0.10 ± 0.03	*0.15 ± 0.03
TCD4 +	12.77 ± 1.83	*25.68 ± 2.65	*22.02 ± 3.07	*38.75 ± 2.16
TCD8 +	6.74 ± 0.88	*13.66 ± 1.1	*13.16 ± 2.31	*20.23 ± 1.94
CD4 + /CD8 +	1.85 ± 0.20	1.79 ± 0.33	1.68 ± 0.31	1.92 ± 0.10
CD8 + , CD28 + , CD57- (naive)	28.30 ± 1.10	*36.51 ± 2.92	*42.21 ± 3.57	*50.71 ± 1.18
CD4 + , CD28 + , CD57- (naive)	30.37 ± 1.45	*41.52 ± 1.57	*43.53 ± 1.68	*58.54 ± 2.02
CD8 + , CD57 + (aged)	20.79 ± 0.74	*16.54 ± 1.26	*14.98 ± 1.97	*13.42 ± 1.09
CD4 + , CD57 + (aged)	23.19 ± 1.7	21.06 ± 1.57	21.92 ± 1.08	*19.46 ± 2.88
CD8 + CD28 + CD57 + (naïve aged)	22.09 ± 1.13	*16.70 ± 1.01	*18.23 ± 1.95	*17.58 ± 1.15
CD8 + , CD28 -, CD57 + (Memory aged)	42.90 ± 0.97	*37.96 ± 1.07	*36.28 ± 1.64	*28.98 ± 0.88
CD4 + , CD28 + , CD57 + (naïve aged)	28.33 ± 1.2	*20.61 ± 0.9	*20.97 ± 1.99	*19.57 ± 1.05
CD4 + , CD28 -, CD57 + (Memory aged)	45.4 ± 0.45	*39.68 ± 2.27	*37.96 ± 2.75	*35.21 ± 0.78

Additionally, there was an increase in indices of TCD4 + , TCD8 + , pristine TCD4 + (CD4 + , CD28 + , and CD57-), and naive TCD8 + (CD8 + , CD28 + , and CD57-) compared to the control group. Other results indicated a significantly lower level of the indicators of thymus atrophy and TCD4 + (CD4 + , CD57 +) only in the combined training group compared to the control group. There was also no difference in the ratio of CD4 + to CD8 + across the groups.

## Discussion

The results of the present study, conducted to investigate the impact of three types of water-based training on immune system aging indicators and thymus atrophy, revealed that only the combined training group demonstrated a significant reduction in thymus weight (reduction of atrophy) compared to the control group. Woods et al. (2003) investigated thymus atrophy in aged rats and reported that 16 weeks of endurance training on a treadmill did not affect thymus weight. Meneguello-Coutinho et al. (2014) reported no difference in the thymus weight of elderly rats between the endurance water-based training and control groups. In this study, elderly rats underwent a dietary program and aerobic swimming exercise for 6 weeks, swimming five days a week for one hour in water at a temperature of 30 degrees (Meneguello-Coutinho et al. 2014). These support the findings of the present study regarding endurance training. No studies were found investigating resistance training or combined exercises.

Production of T cells correlates with the mass of thymus epithelial tissue and atrophy of this organ results in a notable decline in the capacity to generate naïve T cells. The age-related changes in thymus tissue involve a decrease in size, fat tissue accumulation, loss of natural tissue structure, and diminished production and maturation of naïve T lymphocytes (Barbouti et al. 2020), ultimately leading to the weakening of immune system in older age. Hence, exercise training, particularly combined training protocols, can significantly impact the critical factors of the immune system by leveraging the benefits of both resistance and endurance training (Meneguello-Coutinho et al. 2014). Overall, additional research is necessary to ascertain the future impact of sports training on thymus atrophy given the limited number of studies and the need for more conclusive findings.

The results of the present study also demonstrated that all three training methods led to increased TCD4 + and TCD8 + indicators compared to the control group. In line with this, Abd El-Kader and Al-Shreef (2018) reported an increased level of CD4 + and CD8 + indices in elderly individuals following 6 months of aerobic and resistance training. However, Xie et al. (2019) found that six weeks of medium and high-intensity swimming training did not improve TCD4 + and TCD8 + indices in mice aged 6 to 8 weeks with multiple sclerosis (Xie et al. 2019). The conflicting results are likely due to differences in the subject type and age of the animals. The rats in this study were elderly (24 months old), whereas young rats were involved in the study by Xie et al. During exercise oxygen consumption can increase up to 10 times leading to a dramatic rise in free radicals. With these changes, the immune system gains a more remarkable ability to combat harmful free radicals in the blood by producing antioxidant enzymes such as superoxide dismutase, catalase, and glutathione. This process regulates antioxidant enzyme activity, boosts cellular immune response, and increases TCD4 + and TCD8 + indicators (Barbouti et al. 2020).

A decrease in the CD4 + /CD8 + index ratio is typically associated with advancing age and is commonly viewed as a reliable indicator of immunity. This ratio can also serve as an indicator for chronic diseases linked to low-grade systemic inflammation. In the present study, the CD4 + /CD8 + index ratio showed no difference between the training and control groups which are in line with that of Bartlett and Duggal (2020). Some other studies in older adults produced conflicting results. Despeghel et al. reported an increased CD4 + /CD8 + index ratio after six weeks of combined exercise (Bartlett and Duggal 2020) While. Abd El-Kader and Al-Shreef (2018) noted a decrease in the CD4 + /CD8 + index following separate aerobic and resistance exercise protocols.

The variations in results may be attributed to factors such as gender, age, nutrition, type of study subjects, and the nature and intensity of training (Alberton et al. 2019). Additionally, the absence of a significant change in the CD4 + /CD8 + index may be linked to the rise in both TCD4 and TCD8 indexes resulting from the exercises conducted in this study. Generally, the characteristics of intense and adequately voluminous exercise promote the expression of interleukin 2, crucial for T lymphocyte proliferation and enhanced cytotoxic activity of natural killer (NK) cells, leading to the elimination of virus-infected cells. Moreover, the collective benefits of fat reduction and improved cardiovascular fitness through exercise might indirectly enhance the immune response by boosting the circulation of immune cells between lymphoid tissues and peripheral organs, thereby reducing the release of stress-related hormones linked to immunosuppression. This process serves to fortify and enhance T lymphocyte communication (Brito-Neto et al. 2019).

The present study’s findings revealed a notable increase in the CD8 + and CD4 + indices of naïve T lymphocytes following 10 weeks of endurance, resistance, and combined training. These outcomes align with previous studies by Bartlett and Duggal (2020) and Woods et al. (2003). On the other hand, a study by Cao Dinh et al. (2019) investigating the impact of 6 weeks of two-intensity resistance training on naïve lymphocytes in older adults revealed no difference in either group after the training period (Bartlett and Duggal 2020). Similarly, Despeghel et al. (2021) found no difference in the indices of naïve T lymphocytes following a 6 week program of low-intensity combined training involving elderly individuals. The disparity of findings across the studies is largely attributable to differences in the duration of the training period and the characteristics of the experimented subjects. The recent research involved a 10-week training regimen, whereas the previous studies focused on six weeks of training on human samples. Additionally, it is worth noting that the current study took place in an aquatic environment.

Due to the principles of hydrodynamics influencing water-based exercises, the drag force can become more prominent, leading to increased resistance in multiple directions during a session (Alberton et al. 2019). Another factor to consider is the ambient temperature, which, as per observations, can significantly impact people's immune systems (Lee, et al. 2019). In this study, the water temperature was maintained at a constant 30 °C as monitored by a thermometer throughout the research period. In the conducted studies, insufficient attention was given to the temperature component and its impact on the immune system. Moreover, Gustafson et al. (2021) identified a correlation between Becker's TCD8 + and VO2max. Thus, enhancing aerobic capacity in the elderly may lead to an increase in naive cells. A notable feature of T lymphocyte aging is the decline in its proliferative capacity, which is closely associated with the expression of CD57 on the surface of T cells (Nguyen et al. 2017), as well as the shortening of telomere length and the reduction in telomerase activity (Pera et al. 2015).

Compared to CD28 + and CD57 − markers, CD57 + markers cannot replicate after antigen stimulation in vitro and are highly susceptible to apoptosis triggered by activation (Yu and Zheng 2019). Additionally, Silva et al. (2016) found that seniors aged 65–85 who engaged in regular physical activity exhibited extended telomere lengths in TCD8 + lymphocytes (Silva et al. 2016). This increase in telomere length serves as a mechanism that hinders the aging process of T lymphocytes. In line with this, the findings of the present study revealed that all three water-based training methods, when compared to the control group, led to a reduction in the markers associated with aged TCD8 + lymphocytes (CD8 + , CD57 +), markers linked to naive TCD8 + lymphocytes (Aged CD8 + , CD28 + , and CD57 +), and memory TCD8 + lymphocytes (CD8 + , CD28-, and CD57 +). Additional markers related to naive TCD4 + lymphocytes (CD4 + , CD28 + , and CD57 +) and memory TCD4 + lymphocytes (CD4 + , CD28-, and CD57 +) also exhibited signs of aging.

Though the markers associated with TCD4 + lymphocytes in older people (CD4 + , CD57 +) exhibited a notable decrease in the combined exercise group compared to the control group, neither endurance nor resistance exercises demonstrated a significant difference. Aging leads to a reduction in naive T lymphocytes and a rise in the build-up of senescent cells, with the most significant changes observed in TCD8 + cells (Fletcher et al. 2005). The findings of this study regarding the markers associated with aging in TCD8 + lymphocytes and naive TCD8 + cells are consistent with those of Cao Dinh et al. 2019 study. In this study, which explored the impact of moderate and high-intensity resistance training on T lymphocyte subsets in older women, a decrease in mature TCD8 + and naive TCD8 + cells were noted. Conversely, Despeghel et al. (2021) found no notable variation in the markers of mature TCD8 + lymphocytes following six weeks of combined exercise in a dry environment.

The discrepancy in the results is likely attributed to the variation in the exercise environment. The current study's training took place in water, where unique properties like traction force, homeostatic force, osmolarity, and water temperature can elicit distinct effects on the body compared to land-based training (Alberton et al. 2019). Another factor contributing to the aging of the immune system is the disruption in microRNA regulation of T lymphocyte markers. For instance, there is a significant decrease in the expression of miR-92a in the CD8 + marker during aging, which is also linked to the decline in the CD8 + marker. It has been suggested that the progressive decrease in miR-92a expression may be caused by the declining numbers of naive T cells due to repeated stimulation (Ohyashiki et al. 2011). Zhang et al. (2017) reported that walking on a treadmill at one meter per second for five months enhanced miR-92a gene expression in elderly individuals (69 years old) (Zhang et al. 2017). Consequently, it can be inferred that improving this gene through physical activities, particularly combined exercises that encompass both endurance and resistance training, and increasing telomere length can ameliorate immune system aging.

Finally, the relationship between exercise and thymic atrophy in aging represents a critical area of study that investigates how physical activity can influence immune function. The thymus gland, which is responsible for T cell maturation, experiences substantial atrophy with advancing age, resulting in a decline in the production of naive T cells. Research conducted by Nikolich-Žugich (2018) highlighted that this atrophy is associated with an increase in memory T cells alongside a reduction in the generation of naive T cells, which are essential for mounting effective immune responses to novel pathogens(Nikolich-Žugich 2018). Additionally, a study conducted in 2016 demonstrated that older adults who regularly participated in aerobic exercise exhibited enhanced populations of naive T cells, suggesting that exercise may mitigate thymic atrophy and support immune competence in aging individuals(Duggal et al. 2018).

Comparatively, research conducted by Sellami et al. (2018) further substantiates these findings by elucidating the cellular mechanisms underlying exercise-induced alterations in the thymus. The study identified that exercise elicits a variety of hormonal responses, including elevated levels of thymosin β4, which contributes to the maintenance of thymic structure and function. In contrast, sedentary lifestyles are associated with accelerated thymic involution (Sellami et al. 2018), as indicated by the research of Bartlett and Duggal (2020), which demonstrated that physical inactivity correlates with an increased proportion of memory T cells and a reduction in naive T cells. These divergent outcomes highlight the potential of exercise as a modifiable lifestyle factor that can impact immune aging by preserving thymic function(Bartlett and Duggal 2020 Apr 27)..

Moreover, the studies collectively illuminate a paradox in which, despite the increase in memory T cells associated with aging, the maintenance of a robust naïve T cell repertoire is essential for long-term immune health. Exercise seems to enhance this repertoire, thereby improving the body's capability to confront novel pathogens. Papp et al. (2021) demonstrated that physically active lifestyles correlate with a healthier immune profile, asserting that regular exercise not only mitigates the effects of thymic atrophy but also influences the balance between naïve and memory T cells. This research underscores the potential for exercise to function as a non-pharmacological intervention aimed at improving immune functions in the aging population, thus providing a promising avenue for further investigation (Papp et al. 2021).

However, it is imperative to conduct human studies to draw accurate and valid conclusions. One of the strengths of this research lies in the implementation of exercises within an aquatic environment, which facilitates the simultaneous execution of two types of resistance and aerobic exercises. Furthermore, this study offers a comparative analysis with traditional endurance and resistance exercises. The results demonstrate that the synergistic effects of the combined exercise modalities have elicited significant improvements in the cellular immune system of aged rats. Nevertheless, a notable limitation of this research is the insufficient investigation into the neurotrophic and hormonal factors that may influence the cellular mechanisms involved. Additionally, the absence of a healthy young control group, as well as comparable groups in a terrestrial environment, hinders a comprehensive evaluation of the effects of age and varying training environments on the components of the immune system. Future studies that address these two aspects will yield more precise and meaningful insights.

## Conclusion

While age-related alterations in thymus function have been strongly linked to the immunosenescence phenomenon, the advancement in better understanding of the underpinning molecular mechanisms and interventional strategies have been limited. Results of the present research suggests aquatic aerobic and resistance training can generate favourable effects on thymus function in elderly rats by reducing the Senescence of the cellular immune system. Future human studies are needed to support these results and the implementation of aquatic exercises to elderly people.

## Data Availability

The data that support the findings of this study are not openly available due to reasons of sensitivity and are available from the corresponding author upon reasonable request.
